# P-TDHM: Open-source portable telecentric digital holographic microscope

**DOI:** 10.1016/j.ohx.2024.e00508

**Published:** 2024-01-26

**Authors:** Lei Jin, Ziyang Yu, Aaron Au, Peter Serles, Nan Wang, Jeremy T. Lant, Tobin Filleter, Christopher M. Yip

**Affiliations:** a Institute of Biomedical Engineering, 164 College St, University of Toronto, Toronto, ON M5S 3G9, Canada; b Donnelly Centre for Cellular & Biomolecular Research, University of Toronto, 160 College Street, Toronto, Ontario M5S 3E1, Canada; c Department of Mechanical and Industrial Engineering, 5 King's College Road, Toronto, ON M5S 3G8, Canada; d Civil and Environmental Engineering, 527 College Avenue, Cornell University, Ithaca, NY 14853, United States; e Department of Chemistry, 80 St. George Street, Toronto, ON M5S 3H6, Canada; f Department of Chemical Engineering & Applied Chemistry, 200 College St, Toronto, ON M5S 3E5, Canada; g Department of Biochemistry, University of Toronto, 1 King's College Circle, Toronto, ON M5S 1A8, Canada

**Keywords:** Portable telecentric digital holographic microscopy, Holographic microscopy, Quantitative phase imaging, Portable microscope

## Abstract

We present the design of a low-cost, portable telecentric digital holographic microscope (P-TDHM) that utilizes off-the-shelf components. We describe the system’s hardware and software elements and evaluate its performance by imaging samples ranging from nano-printed targets to live HeLa cells, HEK293 cells, and *Dolichospermum* via both in-line and off-axis modes. Our results demonstrate that the system can acquire high quality quantitative phase images with nanometer axial and sub-micron lateral resolution in a small form factor, making it a promising candidate for resource-limited settings and remote locations. Our design represents a significant step forward in making telecentric digital holographic microscopy accessible and affordable to the broader community.

## Highlights

• In comparison to commercially available DHM systems, P-TDHM is fully modular, allowing users to replace and customize individual modules to meet their specific experimental requirements.
• The telecentric design removes the need for complex optics.
• Users can easily switch between in-line and off-axis modes of P-TDHM, depending on their specific experimental needs.

## Graphical abstract

**Figure:**
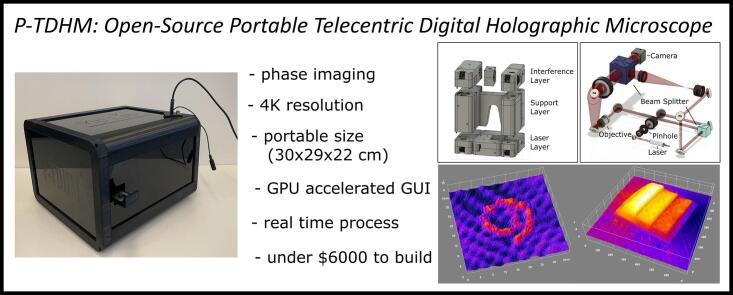

## Specifications table

**Table:** 

Hardware name	*Portable Telecentric Digital Holographic Microscopy*
Subject area	• Engineering and materials science • Biological sciences (e.g., microbiology and biochemistry)
Hardware type	Imaging tools
Closest commercial analog	*HoloMonitor*
Open source license	*CC BY-NC-SA*
Cost of hardware	*$6000 USD*
Source file repository	*https://zenodo.org/record/8206220*

## 1 Hardware in context

Digital holographic microscopy (DHM) is a bright field imaging technique that uses a single exposure to obtain a three-dimensional (3D) quantitative phase image [1]. Its high temporal resolution, limited only by the camera acquisition speed, has had an enormous impact in the fields of biology [2], [3] and micro-electromechanical systems (MEMS) [4], [5]. In-line DHM utilizes diffractive propagation methods for real-time particle [6] and cell tracking [7]. On the other hand, off-axis DHM is well-suited for real-time monitoring of sample dynamics [8], [9]. Telecentric digital holographic microscopy (TDHM) is an extension of DHM that compensates for spherical aberration in the optical objectives by adding a tube lens, applicable to both in-line and off-axis DHM. This design optically mitigates high-order phase aberrations in the hologram[18], effectively reducing wavefront distortions introduced by the microscope objective [10], [11], [12]. It bypasses the computation cost and image quality deterioration through the elimination of numerical compensations, such as polynomial fitting [19].

Current commercial off-axis DHM systems are expensive (> $30000 USD) and bulky (benchtop size). Systems such as the T-1000 (LynceeTec Inc.) and HO-DHM-UT01 (Holmarc, Inc.) incorporate piezo stages and control boxes to optimize interference fringes and suppress coherent noise [13]. However, these electromechanical devices increase system complexity and contribute to their high cost, which can be a barrier to use. The HoloMonitor M4 (PHI Inc.) is a phase imaging system small enough (310 x 180 x 85 mm) to fit in an incubator [14]. However, its portability comes into question for in situ monitoring as it requires the use of various peripherals, such as a laser driver, during operation [15]. Additionally, the HoloMonitor’s non-telecentric design limits its lateral resolution, making it less suitable for high-resolution applications [16], [17].

**Fig. 1:**
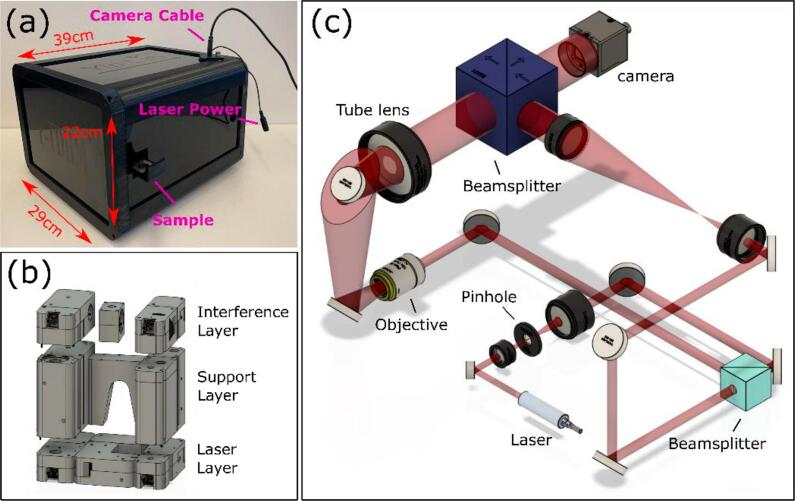
Configuration of the proposed P-TDHM. (a) is the picture of the proposed system enclosed by acrylic panels, comprising three distinct layers (b). The system light path is shown in (c).

This paper presents a portable TDHM (P-TDHM) for imaging of transparent and translucent samples containing both in-line and off-axis functionalities (Fig. 1), with a supporting software pipeline. With the help of a mathematical optimization model [20], we simplified the optics construction and eliminated the need for electromechanical systems for hologram optimization. As a result, we have now developed a customizable DHM design that incorporates 3D-printed parts and features a user-friendly Graphical User Interface (GUI).

## 2 Hardware description

The system is comprised of three layers, each housing specific optical components, as shown in Fig. 1. The laser and interference layers manipulate and modulate the laser beam into interference fringes, respectively. The support layer incorporates an optical path-matching mechanism to compensate for objective-induced spherical aberrations by matching the focal length of the tube lens. Using reconstruction algorithms [21], P-TDHM can digitally refocus on the target without the need for physical adjustments [22]. The design allows for an x-y stage should that be needed for sample positioning. As currently designed, P-TDHM has dimensions of 30 × 29 × 22 cm and weighs 5.2 kg, including its casing. The P-TDHM can switch between in-line and off-axis operation through the use of a beam block slider.

P-TDHM can achieve sub-wavelength axial resolution, depending on the signal-to-noise ratio of the camera and sample clarity. The system's theoretical lateral resolution is determined by the numerical aperture of the objective lens and the laser wavelength. The resulting field of view only depends on the magnification and the sensor size because the optic field is greater than that of the camera sensor. Based on our hardware setup, the user can replace the objective with other objectives as necessary to meet the image requirements of the experiment, as seen in Table 1.

**Table 1:** Objectives with respective theoretical lateral resolution and field of view.

Objectives (Magnification / NA)	Lateral Resolution (μm)	Field of View (μm)
4X / 0.1	3.9	1387 x 1850
10X / 0.25	1.6	555 x 740
20X / 0.35	1.2	277 x 370
20X / 0.75	0.52	277 x 370
60X / 0.9	0.44	92 x 133
100X / 0.95	0.41	55 x 74

P-TDHM uses a single exposure to generate 3D phase maps or 3D volumes for position tracking and imaging. As such, the acquisition speed is solely dependent on the camera sensor. The camera can be replaced to achieve the desired speed and sensor resolution [20]. P-TDHM uses a custom software application developed using PyQt5 in Python 3 to provide users with a graphical interface for adjusting the DHM parameters as well as viewing, processing, and saving images. The software is based on a series of extensible Standard Operation Procedures (SOPs) developed for the in-line and off-axis modes of P-TDHM. P-TDHM is fully customizable and 3D-printable, which significantly reduces material expenditure and enhances the system's flexibility. Given its portability and flexibility, P-TDHM is ideally suited for field studies and on-site imaging.

Below is the specification of P-TDHM system:

**Table:** 

P-TDHM Parameters	Details
Dimensions	30 cm × 29 cm × 22 cm
Camera (BFS-U3-120S4M-CS)	Pixel size:1.85 μm, Frame rate: 31 fps, Resolution:4000 x 3000, ADC: 10-bit, 12-bit
Objective (magnification / NA)	Olympus SLMPlan 20x / 0.35
Resolution	Lateral: 1.2 μm, Axial: 50 nm
Field of View Dimensions	277 × 370 μm
Cost of Hardware	$6000

## Design files

### Design files summary

**Table:** 

**Design file name**	**File type**	**Open source license**	**Location of the file**
*1st_layer_Beam_spliter*	3D Model(.stl)	*CC BY-NC-SA*	*https://zenodo.org/record/8206220*
1st_layer_Beam_support	3D Model(.stl)	*CC BY-NC-SA*	*https://zenodo.org/record/8206220*
1st_layer_Laser_module	3D Model(.stl)	*CC BY-NC-SA*	*https://zenodo.org/record/8206220*
1st_layer_Extra_support	3D Model(.stl)	*CC BY-NC-SA*	*https://zenodo.org/record/8206220*
1st_layer_ND_filter	3D Model(.stl)	*CC BY-NC-SA*	*https://zenodo.org/record/8206220*
1st_layer_Obj_mirror	3D Model(.stl)	*CC BY-NC-SA*	*https://zenodo.org/record/8206220*
1st_layer_Objective_holder	3D Model(.stl)	*CC BY-NC-SA*	*https://zenodo.org/record/8206220*
1st_layer_Ref_mirror	3D Model(.stl)	*CC BY-NC-SA*	*https://zenodo.org/record/8206220*
1st_layer_Sample_holder	3D Model(.stl)	*CC BY-NC-SA*	*https://zenodo.org/record/8206220*
1st_layer_Pinhole_adapter	3D Model(.stl)	*CC BY-NC-SA*	*https://zenodo.org/record/8206220*
1st_layer_Cube_adapter	3D Model(.stl)	*CC BY-NC-SA*	*https://zenodo.org/record/8206220*
1st_cover_Beam_splitter	3D Model(.stl)	*CC BY-NC-SA*	*https://zenodo.org/record/8206220*
1st_cover_Laser_module	3D Model(.stl)	*CC BY-NC-SA*	*https://zenodo.org/record/8206220*
1st_cover_ND_filter	3D Model(.stl)	*CC BY-NC-SA*	*https://zenodo.org/record/8206220*
1st_cover_Obj_mirror	3D Model(.stl)	*CC BY-NC-SA*	*https://zenodo.org/record/8206220*
1st_cover_Objective_holder	3D Model(.stl)	*CC BY-NC-SA*	*https://zenodo.org/record/8206220*
1st_cover_Ref_mirror	3D Model(.stl)	*CC BY-NC-SA*	*https://zenodo.org/record/8206220*
2nd_layer_Beamspander	3D Model(.stl)	*CC BY-NC-SA*	*https://zenodo.org/record/8206220*
2nd_layer_Cam_Cube	3D Model(.stl)	*CC BY-NC-SA*	*https://zenodo.org/record/8206220*
2nd_layer_mirror	3D Model(.stl)	*CC BY-NC-SA*	*https://zenodo.org/record/8206220*
2nd_layer_ND_filter	3D Model(.stl)	*CC BY-NC-SA*	*https://zenodo.org/record/8206220*
2nd_layer_Obj	3D Model(.stl)	*CC BY-NC-SA*	*https://zenodo.org/record/8206220*
2nd_layer_support_ND	3D Model(.stl)	*CC BY-NC-SA*	*https://zenodo.org/record/8206220*
3rd_layer_Beamspander_back	3D Model(.stl)	*CC BY-NC-SA*	*https://zenodo.org/record/8206220*
3rd_layer_Beamspander_BackCover	3D Model(.stl)	*CC BY-NC-SA*	*https://zenodo.org/record/8206220*
3rd_layer_Beamspander_front	3D Model(.stl)	*CC BY-NC-SA*	*https://zenodo.org/record/8206220*
3rd_layer_Cam_cube	3D Model(.stl)	*CC BY-NC-SA*	*https://zenodo.org/record/8206220*
3rd_layer_ND_filter	3D Model(.stl)	*CC BY-NC-SA*	*https://zenodo.org/record/8206220*
3rd_layer_Top_mirror	3D Model(.stl)	*CC BY-NC-SA*	*https://zenodo.org/record/8206220*
3rd_layer_Tube_lens	3D Model(.stl)	*CC BY-NC-SA*	*https://zenodo.org/record/8206220*
3rd_layer_TubeLens_cover	3D Model(.stl)	*CC BY-NC-SA*	*https://zenodo.org/record/8206220*
3rd_cover_cube	3D Model(.stl)	*CC BY-NC-SA*	*https://zenodo.org/record/8206220*
3rd_cover_mirror	3D Model(.stl)	*CC BY-NC-SA*	*https://zenodo.org/record/8206220*
3rd_cover_NDfilter	3D Model(.stl)	*CC BY-NC-SA*	*https://zenodo.org/record/8206220*
3rd_cover_tube	3D Model(.stl)	*CC BY-NC-SA*	*https://zenodo.org/record/8206220*
Box_Bot_long_down	3D Model(.stl)	*CC BY-NC-SA*	*https://zenodo.org/record/8206220*
Box_Bot_long_up	3D Model(.stl)	*CC BY-NC-SA*	*https://zenodo.org/record/8206220*
Box_Bot_short_left	3D Model(.stl)	*CC BY-NC-SA*	*https://zenodo.org/record/8206220*
Box_Bot_short_right	3D Model(.stl)	*CC BY-NC-SA*	*https://zenodo.org/record/8206220*
Box_Side_back_left	3D Model(.stl)	*CC BY-NC-SA*	*https://zenodo.org/record/8206220*
Box_Side_back_right	3D Model(.stl)	*CC BY-NC-SA*	*https://zenodo.org/record/8206220*
Box_Side_front_left	3D Model(.stl)	*CC BY-NC-SA*	*https://zenodo.org/record/8206220*
Box_Side_front_right	3D Model(.stl)	*CC BY-NC-SA*	*https://zenodo.org/record/8206220*
Box_Top_long_up	3D Model(.stl)	*CC BY-NC-SA*	*https://zenodo.org/record/8206220*
Box_Top_long_down	3D Model(.stl)	*CC BY-NC-SA*	*https://zenodo.org/record/8206220*
Box_Top_short_left	3D Model(.stl)	*CC BY-NC-SA*	*https://zenodo.org/record/8206220*
Box_Top_short_left	3D Model(.stl)	*CC BY-NC-SA*	*https://zenodo.org/record/8206220*
Cover_Cam (acrylic)	3D Model(.stl)	*CC BY-NC-SA*	*https://zenodo.org/record/8206220*
Cover_front (acrylic)	3D Model(.stl)	*CC BY-NC-SA*	*https://zenodo.org/record/8206220*
Cover_ND_side (acrylic)	3D Model(.stl)	*CC BY-NC-SA*	*https://zenodo.org/record/8206220*
Cover_Sample_side (acrylic)	3D Model(.stl)	*CC BY-NC-SA*	*https://zenodo.org/record/8206220*
Cover_Top&Bot (X2) (acrylic)	3D Model(.stl)	*CC BY-NC-SA*	*https://zenodo.org/record/8206220*
BeamBlock_in_line	3D Model(.stl)	*CC BY-NC-SA*	*https://zenodo.org/record/8206220*
BeamBlock_off_axis	3D Model(.stl)	*CC BY-NC-SA*	*https://zenodo.org/record/8206220*
DHMViewer	Compressed Python package (. zip)	GNU General Public License v3 (GPLv3)	*https://zenodo.org/record/8206220*

**Laser module**: The pinhole unit improves the spatial coherence property of the illumination source, and the beam expander increases the field of view.

**ND filter**: The ND filter adjusts the intensity of the object and reference beam, ensuring they are matched to achieve optimal interference contrast [23], [24].

**First layer beam splitter**: The beam splitter divides the illumination beam into the object and reference beams.

**Mirrors**: Mirrors alter the direction of the beam. Together, they utilize vertical space to create a 3D optical path, resulting in a smaller system for portability.

**Sample holder module**: The module holds samples for imaging. Users can redesign this component to meet their experimental requirements. A box of size 1.2″ x 2.3.

x 0.85″ has been reserved for the custom sample holder designs. Please refer to the assembling files for best fit with respect to the optical path. The focus plane of the objective we listed is 6.8 mm away from the objective side.

**Objective holder module**: The module holds microscope objectives (SLMPlan 20x/0.35). Users can customize this part to fit their desired objective.

**Layer cover modules**: The layer covers separate the different layers and allow the modules to function independently. They provide the flexibility for users to redesign each module without affecting other layers.

**Second layer supports**: The second layer provides support to the third layer and maintains the correct focal distance for the tube lens.

**Third layer beam splitter**: The beam splitter merges the object and reference beam, resulting in the superposition of the two beams on the camera sensor. The mirror in the reference path allows precise control of the interference fringe to optimize P-TDHM image quality [20].

**Third layer beam expander back & cover**: The beam expander helps to match the size of the reference beam with the size of the objective beam. The residual spherical aberration caused by the microscope objective can be compensated for using the linear stage.

**Box and acrylic boards:** These components protect the system from its surroundings.

## Bill of materials

### Bill of materials summary

**Table:** 

**Designator**	**Component**	**Quantity**	**Unit cost [USD]**	**Total cost [USD]**	**Source of materials**	**Material type**
SLMPlan 20x/0.35	Objective	1	$1175.00	$1175.00	https://www.spachoptics.com/olympus-slmplan-20x-objective-lens-p/olympus-slmplan-20x.htm	Non-specific
BFS-U3-120S4M-CS	CMOS camera	1	$491.00	$491.00	https://www.flir.ca/products/blackfly-s-usb3/?model = BFS-U3-120S4M-CS	Non-specific
CPS635	Laser diode module	1	$98.45	$98.45	https://www.thorlabs.com/thorproduct.cfm?partnumber = CPS635	Non-specific
BS004	0.5″ beam splitter	1	$183.01	$183.01	https://www.thorlabs.com/thorproduct.cfm?partnumber = BS004	Non-specific
BS031	2″ beam splitter	1	$526.86	$526.86	https://www.thorlabs.com/thorproduct.cfm?partnumber = BS031	Non-specific
AC127-019-A-ML	Beam expander	1	$87.35	$87.35	https://www.thorlabs.com/thorproduct.cfm?partnumber = AC127-019-A-ML	Non-specific
AC254-030-A-ML		1	$116.46	$116.46	https://www.thorlabs.com/thorproduct.cfm?partnumber = AC254-030-A-ML	Non-specific
P50D	50 μm pinhole	1	$73.41	$73.41	https://www.thorlabs.com/thorproduct.cfm?partnumber = P50D	Non-specific
LMR1	Pinhole mount	1	$15.69	$15.69	https://www.thorlabs.com/thorproduct.cfm?partnumber = LMR1	Non-specific
M-DS25-X	Linear stage	1	$124.00	$124.00	https://www.newport.com/p/M-DS25-X	Aluminum
NDC-25C-4 M-A	ND filter	1	$506.90	$506.90	https://www.thorlabs.com/thorproduct.cfm?partnumber = NDC-25C-4M	Non-specific
NDC-50C-2 M-A	ND filter	1	$530.19	$530.19	https://www.thorlabs.com/thorproduct.cfm?partnumber = NDC-50C-2M-A	Non-specific
AC127-019-A-ML	Beam expander	1	$87.35	$87.35	https://www.thorlabs.com/thorproduct.cfm?partnumber = AC127-019-A-ML	Non-specific
AC254-250-A-ML		1	$108.70	$108.70	https://www.thorlabs.com/thorproduct.cfm?partnumber = AC254-250-A-ML	Non-specific
AC508-180-A-ML	Tube lens	1	$151.96	$151.96	https://www.thorlabs.com/thorproduct.cfm?partnumber = AC508-180-A-ML	Non-specific
7T184-13	Linear stage	1	$277.08	$277.08	https://www.standa.lt/products/catalog/translation_rotation?item = 254	Aluminum
KS05	0.5″ mirror mount	4	$80.14	$320.56	https://www.thorlabs.com/thorproduct.cfm?partnumber = KS05	Aluminum
BB05	0.5″ mirror	4	$53.06	$212.24	https://www.thorlabs.com/thorproduct.cfm?partnumber = BB05-E01	Non-specific
U50-PL	1″ mirror mount	5	$118.00	$590.00	https://www.newport.com/p/U50-PL	Aluminum
BB1-E02	1″ mirror	5	$77.35	$386.75	https://www.thorlabs.com/thorproduct.cfm?partnumber = BB1-E02	Non-specific
UPA1	1″ mirror adapter	5	$17.30	$86.50	https://www.newport.com/p/UPA1	Non-specific

### 5 Build instructions

All the parts were 3D printed using PLA + filament with 15 % infill and three shells on a RAISE3D Pro2 3D printer using files sliced by IdeaMaker. The layer height was set to 0.2 mm. The support varied depending on the parts themselves (Table Design Files Summary), as printing parameters depend on the 3D printer. The complete assembly file can be found in the online repository (*https://zenodo.org/record/8206220*). The screw types are indicated in the table:

**Table:** 

**Screws**	**Specifications**	**Quantities**
①	#6–32 x 1/2″ UNC Hex Socket Head Cap	90
②	#8–32 x 3/8″ UNC Hex Socket Head Cap	8
③	#8–32 x 1/2″ UNC Hex Socket Head Cap	3
④	M2 x 3 mm Hex Socket Head Cap	6
⑤	M2 x 5 mm Hex Socket Head Cap	6

## Laser Safety Notice

The build includes a Class IIIa laser diode module (4.5 mW, 635 nm). Do not power the laser during assembly. Please wear appropriate PPE during optical alignment.

**Fig. 2:**
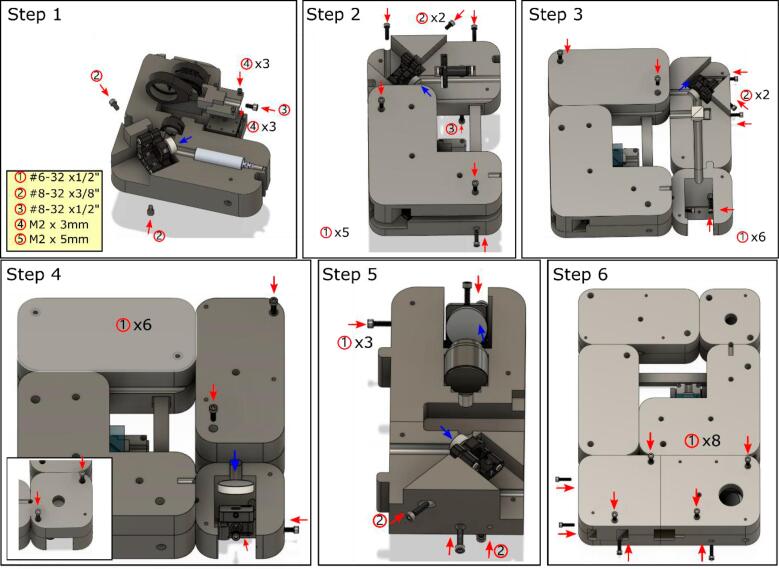
First layer assembly visual guide. The first layer expands the illumination beam size and splits the laser into object beam and reference beam.

First layer assembly (Fig. 2).

1. Mount the laser, pinhole, linear stage (M-DS25-X), mirror, and beam splitter on the printed part (1st_layer_Laser_module.stl)

Alignment: Turn on the laser. Align the laser beam to go through the pinhole by adjusting the linear stage.

2. Cover the laser module with (1st_cover_Laser_module.stl) and mount the mirror and ND filter on (1st_layer_ND_filter.stl). Connect it to the enclosed laser module with (1st_layer_Beam_support.stl).

Connect the (*1st_layer_Beam_spliter.stl and* 1st_layer_Ref_mirror.stl) to the existing body and mount the mirror and the beam splitter

Alignment: Turn on the laser. Align the laser beam to propagate along the optical path and go through the center of the beam splitter.

4. Mount the mirror on (1st_layer_Ref_mirror.stl). Then enclose the body with (1st_cover_Beam_splitter.stl and 1st_cover_Ref_mirror.stl).

5. Mount objective and mirrors on (1st_layer_Obj_mirror.stl and 1st_layer_Objective_holder.stl) separately, then connect them with screws.

6. Mount (1st_layer_Extra_support.stl) on the parts from step 5, then connect to the main body. After aligning the object beam, enclose the first layer.

**Fig. 3:**
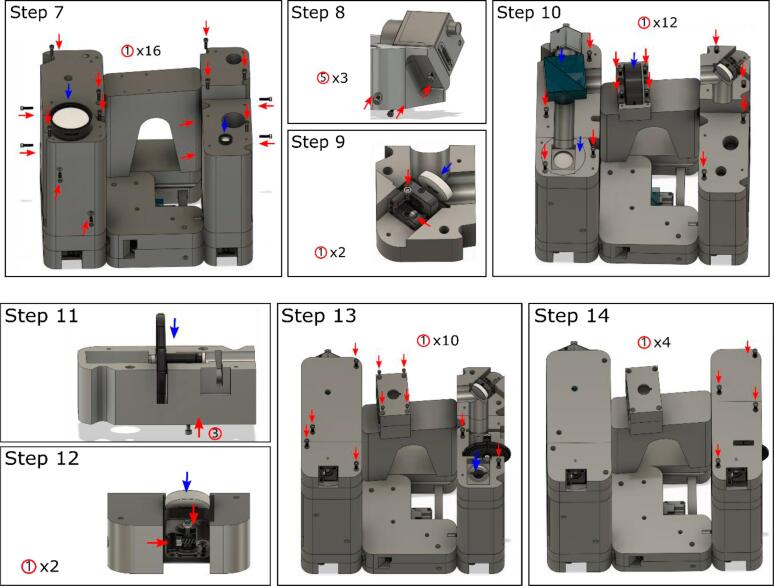
Second and third layer visual guide. The second layer supports the third layer and provides a proper focal distance for the tube lens. The third layer adjusts the interference angle to obtain high quality holograms.

Second and third layer assembly (Fig. 3).

7. Mount the second layer parts, shown in the design files summary, on the first layer individually and install the lens (AC127-019-A-ML, AC508-180-A-ML).

8. Mount the FLIR camera (3rd_layer_Cam_cube.stl).

9. Mount the reference mirror (3rd_layer_Top_mirror.stl).

10. Mount most parts of the third layer on the existing body, including the 2″ beam splitter (BS031), mirror, and 2″ lens (AC254-250-A-ML).

11. Mount the ND filter (3rd_layer_ND_filter.stl).

12. Mount the mirror (3rd_cover_tube.stl).

13. Enclose the object beam and beam splitter modules. Turn on the laser and camera, aligning the object beam located in the center of the camera. Mount the ND filter on the main body (3rd_layer_ND_filter.stl).

14. Enclose the reference beam path. Turn on the laser and camera, adjusting the manual stage and reference mirror until the desired Fourier spectrum is obtained

**Fig. 4:**
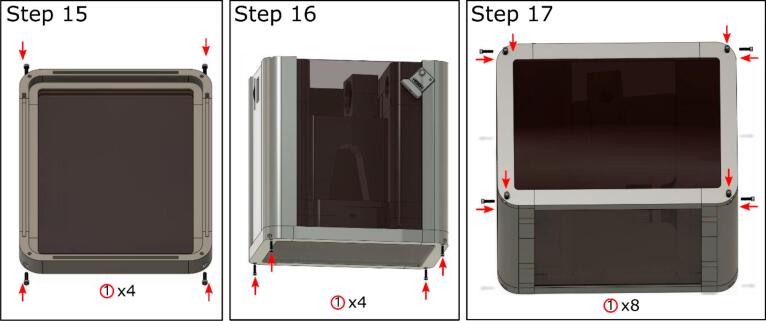
Coverage visual guide. The coverage isolates the optic system from the environment.

Coverage assembly (Fig. 4).

15. Mount the parts together with the black acrylic boards. (Box_Bot_x.stl)

16. Connect to the main body and mount the parts with black acrylic boards. Be careful to avoid damaging the laser cable when mounting the side piece near the camera. (Box_Side_x.stl).

17. Enclose the instrument using (Box_Top_x.stl) and the acrylic board

Routine Maintenance

Due to vibration and use, P-TDHM may occasionally need to be re-aligned. Alignment requires opening the external panels to reach the mirror mounts at the corners. Adjust the ND filters and ensure the intensity of the object beam is approximately twice that of the reference beam [23], [24].

## 6 Operation instructions

**Software Installation and Setup**

System requirements: 64-bit Windows 7 or later, macOS 10.14 or later, Ubuntu Linux 18.04 or later, with Python 3.7 or later, a monitor with 1280x800 or higher resolution, and a computer with a USB3 port.

1. Download and install the latest Python3 (Version 3.7 or above) release. (3.7.13 and 3.8.5 has been tested).

2. Download and extract the package *DHMViewer* into the desired directory.

3. Open a terminal in the *DHMViewer* directory, install the dependencies using pip command. The dependencies include *Matplotlib*, *NumPy*, *Pillow*, *PyQt5*, *Scikit*_*image*, and *Tifffile*.

“pip install -r./configuration.txt”

4. Download and Install the Latest Spinnaker Full SDK from FLIR official website (https://www.flir.ca/support-center/iis/machine-vision/downloads/spinnaker-sdk-and-firmware-download/). We tested SpinnakerSDK_FULL_3.0.0.118_x64.exe and SpinnakerSDK_FULL_2.7.0.128_x64.exe.

**Intended Workflow**

1. Connect the DC power for the laser module and plug the FLIR camera's USB cable into the computer's USB3 port.
2. Select the image mode (in-line or off-axis) by sliding the beam blocker to the respective positions.
3. Launch the *Spinnaker SpinView* software, select camera interface and start capturing.
4. Fine-tune the ND filters to optimize intensity of object and reference beams [23], [24] with the help of the GUI exposure views. The intensity ratio between object and reference beams can be 1 ∼ 1.5.
5. Place the samples on the sample holder properly and slide into the microscope body.
6. Captures the background and hologram images with the capture software manufacturer, saving them to two separate directories.
7. Launch the *DHMViewer* program, set the desired operation mode and optical parameters.
8. Specify the locations of background and holograms files in the *DHMViewer* user interface.
9. Optionally specify a Region of Interest (ROI) to be processed.
10. Choose whether to save the processed image, respective file directory, and image types. Select the range of images to be processed.
11. Process (and save) the images, optionally saving the current system configuration.
12. To obtain reconstructed z-stacks for a given off-axis hologram image, repeat steps (7) to (12) and switch between the off-axis and in-line operation modes.

**Software Operation**

**Fig. 5:**
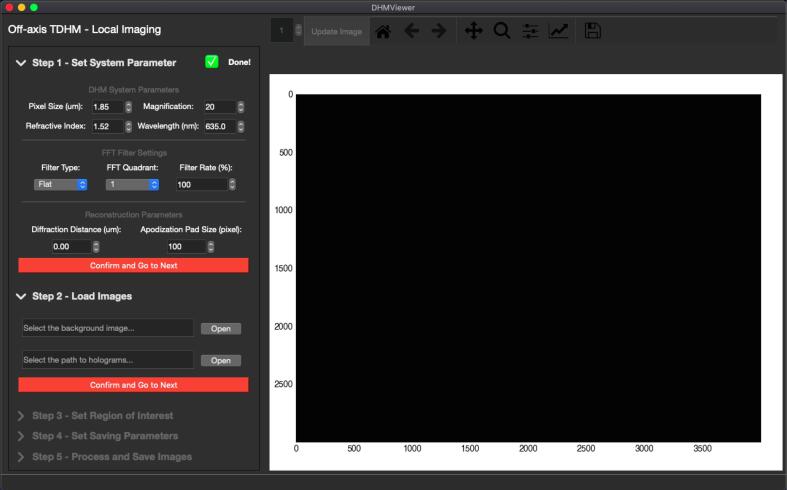
*DHMViewer* main window. The user interface contains a side panel containing SOP items (left), a display and graphics toolbar (top right), and a graphics viewer (right).

**Fig. 6:**
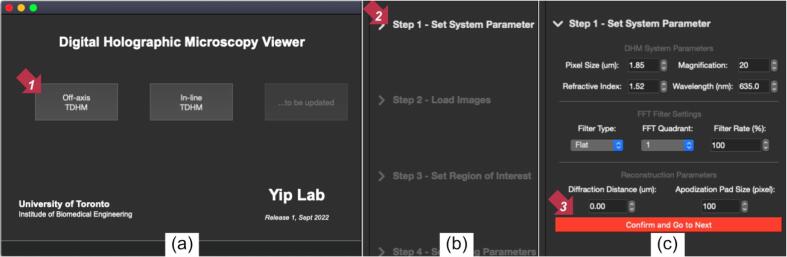
*DHMViewer* menu actions. When the user first launches the program, the launcher window shall appear as in (a). By performing a mouse click action as indicated by the red arrow marked “1″ in (a), the user will observe the SOP of the respective operation mode (”off-axis“) selected in the Main Window. Clicking the dropdown menu pointed by the red arrow ”2″ in (b) results in the expansion of the “*Set System Parameter*” step of the SOP in (c). Clicking the “*Confirm and Go to Next*” button as indicated by the red arrow “3″ will lead to the expansion of the next step (”*Load Images*“ as in Fig. 5.) and the simultaneous closing of the current step (”*Set System Parameter*“). Nevertheless, every previously set dropdown menu can be reopened for setting changes. (For interpretation of the references to colour in this figure legend, the reader is referred to the web version of this article.)

Launch the *DHMViewer* program by double clicking dhm_viewer.py or running the Python command in the terminal. (“Python3./dhm_viewer.py”**)** When the launcher window appears, seen in (Fig. 6a.), click on the desired mode of operation (off-axis or in-line) to switch to the appropriate SOP mode. The main window should appear (Fig. 5.). From the left side panel, click the dropdown menu to expand the details of each step, and click the red proceed button.

1. Optionally load an “.ini” configuration file from the file menu to jump to step 4 (Fig. 7a.). If needed, click the menu items in the menu bar to switch to another DHM mode.

**Fig. 7:**
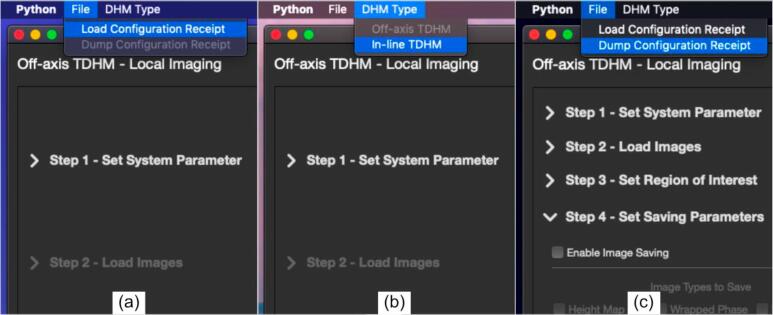
*DHMViewer* program menu items. The “*DHM Type*” mode selector can be used to select the desired SOP for either the in-line or the off-axis mode, visible in (b). The “*Load Configuration Receipt*” menu item is visible in (a) at the start of the execution of a SOP. The additional menu option of “*Dump Configuration Receipt*” is available after all the parameter configurations have been completed, in Step 4 of the SOP, as shown in (c).
2. Set the parameters matching the desired system configuration and camera sensor specification (Fig. 8a.).

**Fig. 8:**
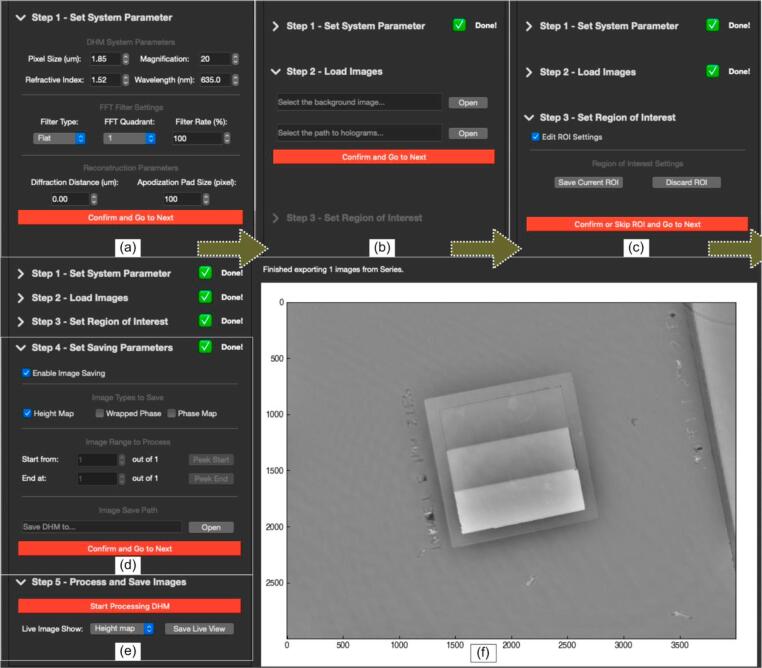
*DHMViewer* graphical user interface, Side panel with the “*Off-axis TDHM*” mode SOP. The “*Set System Parameter*” step of the SOP in (a) includes objective magnification and refractive index. The “*Load Images*” step in (b) consists of input fields and file handling interfaces for selecting the background and hologram image files. The “*Set Region of Interest”* step in (c) employs an interactive rectangular region selection to be performed on the graphics viewer by click-drag action. The “*Set Saving Parameters*” step in (d) includes input fields for saving indices in image sequence, saving image type and file handling interfaces. The “*Process and Save Images*” step in (e) allows format options for processed images to be viewed during processing. (f) illustrates a height map image for a representative target specified in the Image Validation section, displayed in *DHMViewer.*
3. Specify the file location of the background and the directory locations of the hologram image(s). Enter the address by hand or use the file browser by clicking the “*Load*” button. The background and the hologram will be loaded onto the viewer (Fig. 8b.).
4. Optionally specify the Region of Interest. Only the content inside ROI will be processed. Click the checkbox “*Edit ROI*”, then follow the messages above the viewer to select ROI on screen (Fig. 9.). One can also shuffle the spin-box and click the “*Update image*” button at the top of the viewer to view the entire range of images to check the ROI's relative position (Fig. 8c.)

**Fig. 9:**
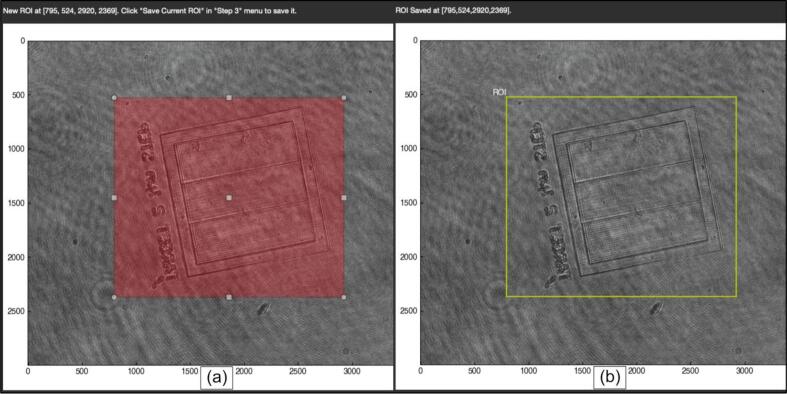
*DHMViewer* graphical user interface, graphics viewer with “*Set Region of Interest*” step in SOP. By enabling the “Enable ROI Settings” checkbox in Fig. 8c., an interactive rectangular region selection can be performed on the graphics viewer by click-drag action in (a). By Clicking the “Save Current ROI” option in Fig. 8c., the ROI is saved and the yellow bounding box in (b) results. Sample: Nano-printed resin target. (For interpretation of the references to colour in this figure legend, the reader is referred to the web version of this article.)
5. Check the “save image” checkbox should the processed images need to be saved. In off-axis mode, select the image type to be saved. Select the range of images to be processed by the start and stop points by the spin-boxes. Click the “peek” button to view the selected start/stop point. Should anything be saved, select a file directory (Fig. 8d.).
6. Click the process image button to process the selected images. A progress bar, a “*Pause*” button, and an “*End task*” button should appear on top of the viewer. Click the respective button to pause the operation or to end the processing and go back to save settings. On the side panel, click the dropdown menu to select the viewing image type during processing; clicking the “*Live save*” button will save the viewer's currently displayed content (Fig. 8e.). In in-line mode, when “*Reconstructed volume*” is selected as the viewing type, each slice of the reconstructed hologram can be viewed after processing and saving is complete.

Once every dropdown menu has been checked, user is able to click save the configuration to save all current settings into a “.ini” file in the desired directory (Fig. 7c.).

## 7 Validation and characterization

**Image validation**

**Fig. 10:**
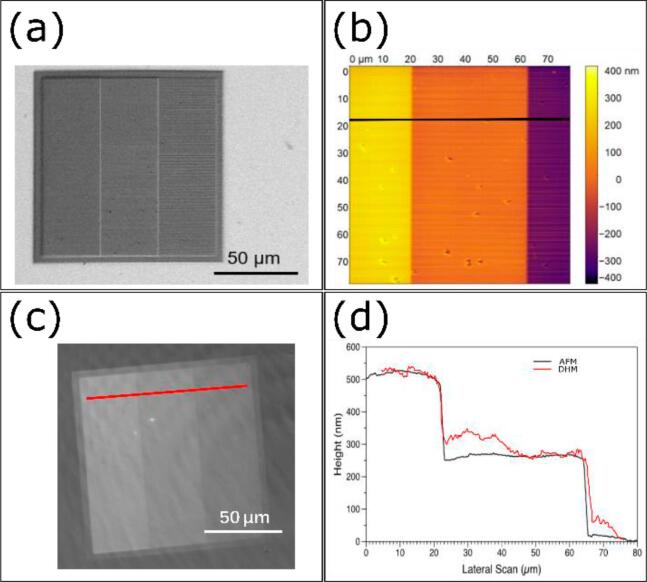
DHM image validation with nano-printed target imaged by (a) SEM, (b) AFM, (c) TDHM. (d) Corresponding section plot of the image result obtained by AFM and TDHM.

The 3D imaging was validated using nano-additive manufacture targets produced by two-photon polymerization (Nanoscribe GmbH, PPGT2) of IP-Dip2 positive photoresist on glass. [25]. After photopolymerization, the polymer has a refractive index of 1.547. The targets are produced using a Nanoscribe Photonic Professional GT2 two-photon polymerization system. This system uses a 100 fs, 80 MHz pulsed laser with a 780 nm wavelength through a 63x focusing objective, which is rastered across the surface in a designated pattern. The target comprised three 40 µm wide, 250 nm high steps. Scanning electron microscope (Hitachi, SU7000) and atomic force microscope (Asylum Research, Cypher) images of the target are shown in Fig. 10a. and 10b, respectively. Fig. 10c. shows the target as imaged by TDHM and Fig. 10d. shows good agreement in both step height and lateral dimensions between the TDHM and AFM images profiles. The result shows that the height detection differences between AFM and P-TDHM was less than 50 nm.

The resulting topography (Optical Path Length (OPL)) is theoretically described by [26],

$$OPL\left(x,\;y\right)=\Phi_{z}\left(x,\;y\right)\cdot \left(n_{s}-n_{m}\right)$$

Where λ is the wavelength of the illumination, $\Phi_{z}\left(x,\;y\right)$ is the thickness distribution of the sample, n_s_ and n_m_ represent the refractive index of the sample and media, respectively.

**3D tracking (in-line mode)**

**Fig. 11:**
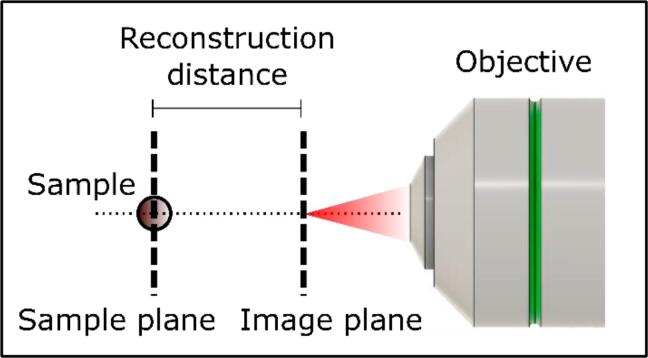
Schematic of in-line reconstruction. The plane which the objective focuses on is called the image plane, and the plane which the target is located is known as the sample plane. Reconstruction is performed using the Angular Spectrum method.

**Fig. 12:**
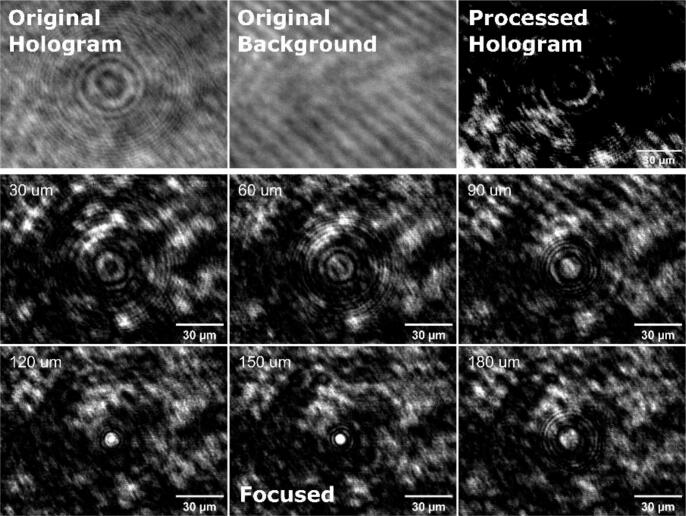
Principle of 3D particle tracking for in-line mode. After subtracting the background, the hologram was reconstructed 200 times with 1 μm steps using the Angular Spectrum method. The focused image was 150 μm away from the image plane.

The in-line beam blocker functions by obstructing the reference beam of P-TDHM thereby establishing the classic in-line TDHM configuration, which is well-suited for volumetric sample tracking. In the absence of the reference beam, the field of view can be regarded as an interference pattern between the original laser field and the image field, which is scattered by the sample. Without the reference field, this configuration offers superior image contrast. To validate its tracking ability, imaging of 4.5 µm diameter polymer particles freely suspended in phosphate-buffered solution (PBS) was performed. As shown in Fig. 11 and Fig. 12, the hologram was generated by subtracting the background from the acquired original image. The Angular Spectrum method [27] was then employed to reconstruct the hologram 200 times at 1 μm increments.

The results of the reconstruction (Fig. 11 and Fig. 12) indicate that the sample plane was determined to be 150 μm away from the image plane. The reconstructed images can be evaluated via its Fourier spectrum with micrometer accuracy [21]. The Angular Spectrum method is employed to reconstruct the entire field of view. Set in in-line mode, P-TDHM system can simultaneously track all particles in 3D with a single-shot hologram, provided the diffraction patterns can be clearly captured.

**Numerical refocusing (off-axis mode)**

**Fig. 13:**
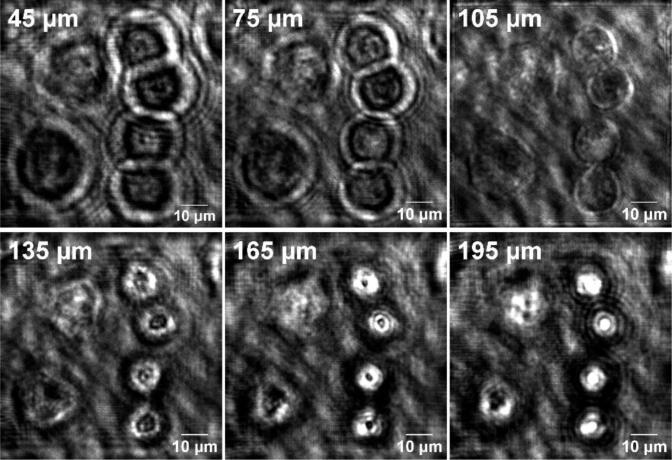
Auto-refocusing results of HeLa cells. With the same reconstruction algorithm used in Fig. 12, the image of HeLa cells was reconstructed six times in different depth positions. This proves that the reconstruction algorithm can also be used to numerically refocus.

The reconstruction algorithm can also be executed in the off-axis mode of the proposed system. Due to the impact of the reference wave, the axial resolution of its reconstruction may not be as high as that of the in-line mode, but it is still sufficient for numerical refocusing of the optic field. As shown in Fig. 13, the unfocused hologram could be reconstructed back into its focus plane numerically.

**Long-Term Imaging**

**Fig. 14:**
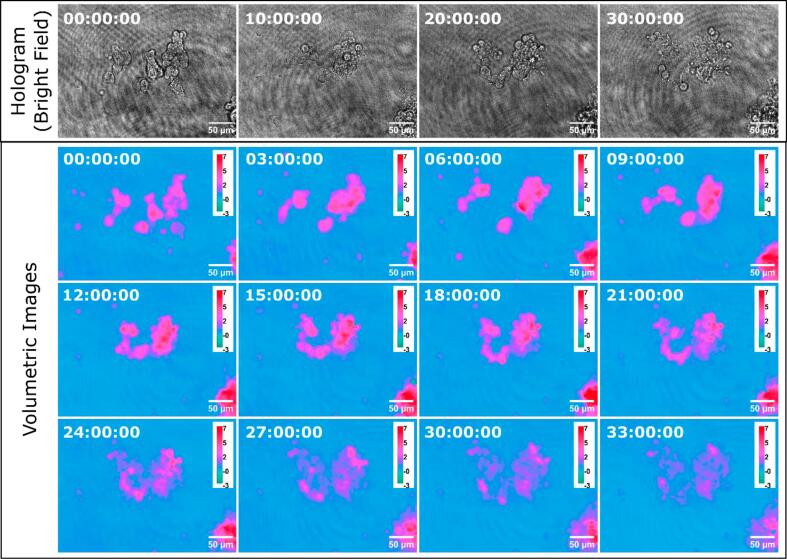
Migration of living HEK239T cells (ATCC CRL-3216) in PBS, imaged by P-TDHM. The entire sequence spans 33 h. The unit of the height bar is micrometer.

To establish the validity of P-TDHM for long term imaging, HEK293 cells were continuously imaged for 33 h. The HEK293T (ATCC CRL-3216) cells were cultured in high glucose (4.5 g/L) Dulbecco's Modified Eagle's medium (Gibco), supplemented with 10 % fetal bovine serum (Gibco) and penicillin/streptomycin (Gibco; 100U/mL penicillin, 100 µg/mL streptomycin). The cells were harvested five hours prior to imaging and transferred into 35 mm culture dishes for further analysis. Following the instructions described previously, the dish with cells was imaged in room temperature directly without adding any extra nutrients, as shown in Fig. 14.

Cell growth and migration continued for 15 h with the nutrient, before the collapse of the aggregation after 20 h. Quantitative phase images (QPI) represent the product of sample thickness and its refractive index, which closely relate to the cell dry mass [28], and is theoretically not impacted by transients in illumination power. Additionally, reconstruction algorithms such as the Angular Spectrum method allow P-TDHM to digitally refocus the images. These attributes suggest that TDHM is better suited for long-term imaging of cellular dynamics than conventional brightfield microscopy.

**In-field Imaging**

Ultimately, the compact design and user-friendly software provides P-TDHM with the in-field imaging capabilities. These distinctive traits collectively point to the potential of P-TDHM as a tool for advancing phytoplankton studies. The significance of monitoring phytoplankton spans industries, research, and natural resource management, as emphasized by [29].

**Fig. 15:**
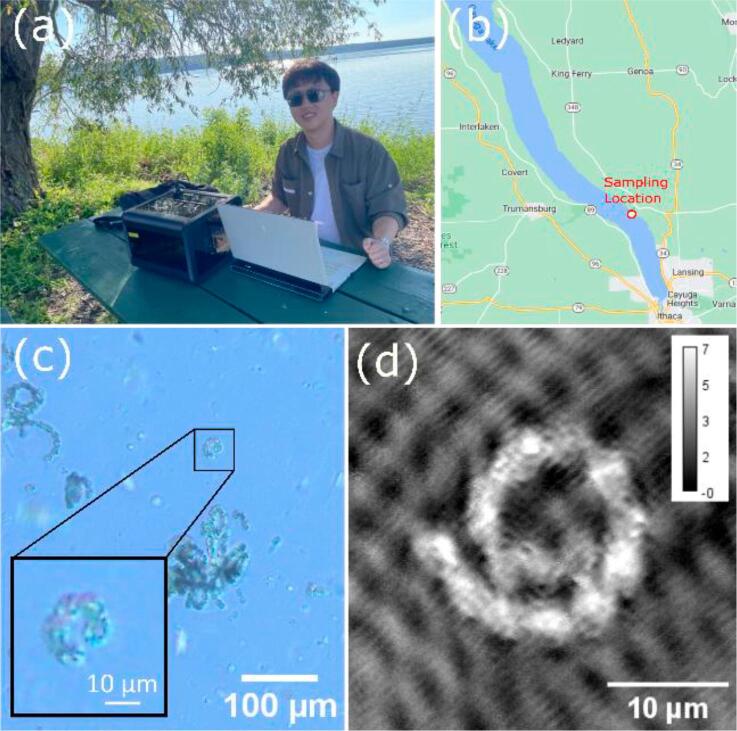
In-field imaging of *Dolichospermum* using P-TDHM. The sample is from Meyers Park in Cayuga Lake, USA. (a) In-field imaging with P-TDHM; (b) sampling location. *Dolichospermum* imaged by (c) bright field and (d) P-TDHM, the unit of the height bar is micrometers.

In a preliminary study, P-TDHM was tested on-site in a remote location.(See Fig. 15) The study was conducted on surface water samples procured during a bloom event on July 11, 2023, along the shoreline of Cayuga Lake, USA. P-TDHM successfully captured detailed images of *Dolichospermum*, the dominant phytoplankton in cyanobacterially harmful algal blooms. This bloom event was later reported by Community Science Institute (CSI), USA, and the dominance of *Dolichospermum* was confirmed [30].

P-TDHM’s inherent stability over fluctuating imaging conditions, such as light sources and focus adjustments, contribute to the method's consistency and reliability in phytoplankton monitoring. Additionally, P-TDHM imaging is not impacted by illumination intensity transients, so it is more reliable to analyze harmful algal blooms from different time and locations. The depth information provided by P-TDHM also offers an additional dimension for more comprehensive analysis.

In-field imaging operations with P-TDHM only require the microscope body and a personal computer. With a custom-designed flow chamber and appropriate algorithms, P-TDHM can be used for particle analysis akin to the FlowCan system (Yokogawa Fluid Imaging Technologies, Inc) with the additional capability of 3D volumetric imaging. Similarly, the user could incorporate waterproof panelling in the design of P-TDHM to perform in-situ, automated and submersible imaging, as demonstrated by FlowCytobot (McLANE Research Laboratories, Inc).

P-TDHM’s inherent flexibility enables many other applications in the field of biology, physics [31], and medical diagnosis [32], [33]. For example, it can be introduced to assist medical diagnosis in blood tests, retinal imaging, and dental imaging. P-TDHM can also be used for airborne particle and aerosol detection to monitor pollutants in air [34], [35]. Additionally, with a proper design of the sample holder with microfluids similar to [36], the proposed system could work as tomographic cytometry as well [37].

## Concluding remarks

The portable telecentric digital holographic microscope (P-TDHM) described herein retains the well-established and comprehensive capabilities of conventional TDHMs but is built in a compact lightweight form factor. Compared to commercial alternatives, the cost-effective nature of P-TDHM confers a high degree of accessibility to a broad range of potential end-users.

**Capabilities**

• High-resolution imaging. The axial resolution of P-TDHM is not limited by conventional diffraction limit.
• Quantitative phase imaging. P-TDHM provides quantitative phase information of the sample, which can be used to measure a sample’s refractive index and thickness.
• Real-time imaging. Volume acquisition is limited only by the acquisition speed of the camera.
• Long-term monitoring. Theoretically, the recording range of the T-DHM is unlimited.
• Portability.

**Limitations**

• Limited imaging depth. P-TDHM requires the illuminating laser to pass through the sample and interfere with the reference beam. As such, the performance of P-TDHM will be limited by the sample’s refractive index and thickness.
• Vibration sensitive. The axial resolution of P-TDHM reaches tens of nanometers, so the environmental vibration would impact the beam interference and the image quality. Thus, users should keep it stable during the imaging, like adding rubber isolation underneath.

## Ethics statements

This work does not involve the use of human and animal subjects.

## CRediT authorship contribution statement

**Lei Jin:** Conceptualization, Formal analysis, Investigation, Methodology, Software, Visualization, Writing – original draft, Writing – review & editing, Validation. **Ziyang Yu:** Software, Investigation, Writing – original draft. **Aaron Au:** Conceptualization, Investigation, Methodology, Resources, Visualization, Writing – review & editing. **Peter Serles:** Investigation, Resources, Writing – review & editing. **Nan Wang:** Data curation, Investigation, Writing – review & editing. **Jeremy T. Lant:** Resources, Writing – review & editing. **Tobin Filleter:** Resources, Supervision. **Christopher M. Yip:** Funding acquisition, Supervision, Writing – review & editing, Resources.

## Declaration of competing interest

The authors declare that they have no known competing financial interests or personal relationships that could have appeared to influence the work reported in this paper.
